# Results of a single-arm pilot study of ^32^P microparticles in unresectable locally advanced pancreatic adenocarcinoma with gemcitabine/nab-paclitaxel or FOLFIRINOX chemotherapy

**DOI:** 10.1016/j.esmoop.2021.100356

**Published:** 2021-12-23

**Authors:** P.J. Ross, H.S. Wasan, D. Croagh, M. Nikfarjam, N. Nguyen, M. Aghmesheh, A.M. Nagrial, D. Bartholomeusz, A. Hendlisz, T. Ajithkumar, C. Iwuji, N.E. Wilson, D.M. Turner, D.C. James, E. Young, M.T. Harris

**Affiliations:** 1 Guy’s & St Thomas’ Hospital NHS Foundation Trust, London, UK; 2 Imperial College Healthcare NHS Trust, London, UK; 3 Monash Health, Clayton, Australia; 4 Austin Hospital, University of Melbourne, Australia; 5 Royal Adelaide Hospital, Adelaide, Australia; 6 Southern Medical Day Care Centre, Wollongong, Australia; 7 The Crown Princess Mary Cancer Centre, Westmead Hospital, Westmead, Australia; 8 Institut Jules Bordet, Brussels, Belgium; 9 Addenbrooke’s Hospital, Cambridge University Hospitals NHS Foundation Trust, Cambridge, UK; 10 Leicester Royal Infirmary, University Hospitals of Leicester NHS Trust, Leicester, UK; 11 OncoSil Medical Limited, Sydney, Australia; 12 Southern Star Research Pty Ltd, Gordon, Australia

**Keywords:** locally advanced pancreatic cancer, ^32^P microparticles, brachytherapy, safety profile, local disease control rate

## Abstract

**Background:**

Unresectable locally advanced pancreatic cancer (LAPC) is generally managed with chemotherapy or chemoradiotherapy, but prognosis is poor with a median survival of ∼13 months (or up to 19 months in some studies). We assessed a novel brachytherapy device, using phosphorous-32 (^32^P) microparticles, combined with standard-of-care chemotherapy.

**Patients and methods:**

In this international, multicentre, single-arm, open-label pilot study, adult patients with histologically or cytologically proven unresectable LAPC received ^32^P microparticles, via endoscopic ultrasound-guided fine-needle implantation, planned for week 4 of 5-fluorouracil, leucovorin, irinotecan and oxaliplatin (FOLFIRINOX) or gemcitabine/nab-paclitaxel chemotherapy, per investigator’s choice. The primary endpoint was safety and tolerability measured using Common Terminology Criteria for Adverse Events version 4.0. The lead efficacy endpoint was local disease control rate at 16 weeks.

**Results:**

Fifty patients were enrolled and received chemotherapy [intention-to-treat (ITT) population]. Forty-two patients received ^32^P microparticle implantation [per protocol (PP) population]. A total of 1102 treatment-emergent adverse events (TEAEs) were reported in the ITT/safety population (956 PP), of which 167 (139 PP) were grade ≥3. In the PP population, 41 TEAEs in 16 (38.1%) patients were possibly or probably related to ^32^P microparticles or implantation procedure, including 8 grade ≥3 in 3 (7.1%) patients, compared with 609 TEAEs in 42 (100%) patients attributed to chemotherapy, including 67 grade ≥3 in 28 patients (66.7%). The local disease control rate at 16 weeks was 82.0% (95% confidence interval: 68.6% to 90.9%) (ITT) and 90.5% (95% confidence interval: 77.4% to 97.3%) (PP). Tumour volume, carbohydrate antigen 19-9 levels, and metabolic tumour response at week 12 improved significantly. Ten patients (20.0% ITT; 23.8% PP) had surgical resection and median overall survival was 15.2 and 15.5 months for ITT and PP populations, respectively.

**Conclusions:**

Endoscopic ultrasound-guided ^32^P microparticle implantation has an acceptable safety profile. This study also suggests clinically relevant benefits of combining ^32^P microparticles with standard-of-care systemic chemotherapy for patients with unresectable LAPC.

## Highlights

• PanCO is the first prospective study of intratumoural ^32^P microparticles for locally advanced pancreatic cancer (LAPC).
• This single-arm study assessed a novel brachytherapy (^32^P microparticles) combined with standard-of-care chemotherapy.
• Treatment-emergent adverse events attributable to ^32^P microparticle implantation were relatively infrequent.
• Local disease control rate at 16 weeks (82%) and resection rate (20%) suggest a clinical benefit of ^32^P microparticles.
• The results suggest that ^32^P microparticles may address a significant unmet need in patients with unresectable LAPC.

## Introduction

Locally advanced pancreatic cancer (LAPC) accounts for ∼30% of all pancreatic cancer presentations.^1^, ^2^, ^3^ By the time clinical symptoms are evident, most patients have tumour invasion that precludes resection with curative intent. Unresectable LAPC has a poor prognosis with a median survival of ∼13.3 months,^4^ with some reports of survival up to 19 months.^2^^,^^5^, ^6^, ^7^, ^8^

Guidelines recommend first-line chemotherapy or induction chemotherapy with consolidative chemoradiotherapy.^5^^,^^9^, ^10^, ^11^ Recommended combination regimens for patients with good performance status include 5-fluorouracil, leucovorin, irinotecan, and oxaliplatin (FOLFIRINOX), gemcitabine/nab-paclitaxel, or other gemcitabine-based chemotherapy regimens. Combining systemic chemotherapy with radiotherapy for local disease control may improve time to progression of local disease, pain control, performance status, and quality of survival, and in a neoadjuvant setting may convert tumours to resectability.^12^^,^^13^ Improvements in overall survival (OS), however, have been elusive,^6^ and due to the location of the pancreas and risk of radiation damage to surrounding tissues and organs, locally-directed radiotherapy is needed.

Brachytherapy offers the option of delivering radiation directly to the tumour via endoscopic ultrasound (EUS)-guided fine-needle injection,^14^ potentially reducing the risk of collateral damage, and was shown to be clinically feasible in unresectable pancreatic cancer.^15^^,^^16^ Phosphorous-32 (^32^P) microparticles is a novel brachytherapy device, approved for use in unresectable LAPC, that implants the required activity of beta radiation-emitting ^32^P microparticles into pancreatic tumours via EUS guidance to deliver an absorbed dose of 100 Gy to the tumour. In combination with gemcitabine monotherapy in 23 patients with LAPC and metastatic disease, ^32^P microparticles showed acceptable tolerability and feasibility, and a case study from an ongoing study suggested positive efficacy outcomes.^17^^,^^18^ The PanCO study was initiated to assess the safety, efficacy, and feasibility of ^32^P microparticles in combination with current standard-of-care chemotherapy (FOLFIRINOX or gemcitabine/nab-paclitaxel) in patients with unresectable LAPC.^19^ Here we present the final results of the PanCO study.

## Methods

The PanCO study was an international, multicentre, single-arm, open-label pilot study conducted at 10 centres in Australia, Belgium, and the UK (ClinicalTrial.gov ID: NCT03003078). The study was designed and conducted in accordance with ISO 14155, applicable local regulations (including European Directive 2001/20/EC), and with the ethical principles laid down in the Declaration of Helsinki. All appropriate ethics committee approvals were obtained, and all participants gave written informed consent.

### Participants

Adult patients were included in the study if they had histologically or cytologically proven, unresectable LAPC, a target tumour diameter 2-6 cm, and Eastern Cooperative Oncology Group performance status 0-1. Included patients also had to have adequate renal, liver, and bone marrow function, life expectancy of at least 3 months at screening, and were not pregnant and using adequate birth control if of child-bearing potential. Exclusion criteria included: evidence of distant metastases based on computed tomography (CT) scan; evidence of radiographic invasion into the stomach, duodenum, or peritoneum; more than one primary lesion; any previous radiotherapy or chemotherapy for pancreatic cancer; use of any investigational agent within the last 30 days; unacceptable risks for EUS-directed implantation according to the investigator; history of malignancy in the last 5 years; or a known allergy to any of the components of the test device. A full list of inclusion and exclusion criteria is shown in Supplementary Table S1, available at https://doi.org/10.1016/j.esmoop.2021.100356.

### Study interventions

Included patients received ^32^P microparticles (OncoSil Medical, Sydney, Australia) and chemotherapy with either FOLFIRINOX or gemcitabine/nab-paclitaxel. The chemotherapy regimen selection was at the investigators’ discretion and was administered according to the local guidelines. The selected chemotherapy regimen was started within 14 days of the screening visit, and implantation of the ^32^P microparticles was planned in week 4 of the chemotherapy cycles, allowing ≥48 h before and after chemotherapy administration and implantation of ^32^P microparticles. ^32^P activity was calculated from the patient's tumour volume to deliver a 100 Gy absorbed dose.^20^ The ^32^P microparticles were implanted directly into the pancreatic tumour via EUS guidance, using a fine-needle aspiration. Following ^32^P microparticle implantation, chemotherapy was continued according to local practice. ^32^P microparticle localization pattern following implantation was assessed by EUS and Bremsstrahlung single-photon emission CT (SPECT)/CT within 4 h and at 7 days post-implantation.

### Assessments and outcome measures

Patient assessments took place at screening and on the day chemotherapy commenced, each week thereafter until 12 weeks after commencement, at week 16, and then every 8 weeks until progression of the target pancreatic tumour. After progression and the end-of-study visit, medical records of each patient were reviewed at 8-weekly intervals.

The primary endpoint was safety and tolerability as measured by the frequency of treatment-emergent adverse events (TEAEs), graded according to National Cancer Institute Common Terminology Criteria for Adverse Events (CTCAE) version 4.0, from the date of chemotherapy commencement to end of study. TEAEs and laboratory values were assessed at each visit until end of study.

Blood and urine samples were collected on the day of ^32^P microparticle implantation (blood collected before and within 4 h of implantation; urine collected within 4 h of implantation) and at 1, 2, and 3 weeks post-implantation (and at 8 weeks post-implantation for blood sample) to assess non-target exposure to ^32^P in approximately 20 study participants. Radioactivity was measured in blood and urine samples using Wallac 1409 automatic liquid scintillation counters. Any free activity in blood and urine on the day samples were taken was expressed relative to implanted activity.

The lead secondary endpoint was local disease control rate (LDCR) at 16 weeks. LDCR was defined as stable disease, partial response, or complete response in the target tumour. Tumour response was measured by CT scan at screening and at 8-weekly intervals assessed independently by central reader analysis using RECIST version 1.1 and target tumour volume measurement.

Other secondary endpoints included: local progression-free survival (LPFS), defined as the time from enrolment to the date of death or the date of the scan used to determine local tumour progression; PFS at any site; and OS defined as the time from enrolment to death by any cause. LPFS and PFS were assessed with and without censoring resected patients at their last CT scan before surgery. Pain was measured using the 10-point Numerical Rating Scale (NRS). Changes in the level of the tumour marker carbohydrate antigen (CA) 19-9 from baseline were measured according to each site’s standard method. As an exploratory analysis, a ≥50% reduction from baseline values or normalization of CA 19-9 was considered clinically important if baseline levels were >35 U/ml.^21^ Additional exploratory analyses were conducted and included: metabolic tumour response according to [^18^F]2-fluoro-2-deoxy-d-glucose positron emission tomography (^18^F-FDG-PET) measured at baseline and week 12, assessed by the independent central reader for total lesion glycolysis (TLG), and standardized uptake values, SUV_Max_ and SUL_Max_^22^; target tumour volume change assessed by central reader (using Voxels of Interest and eMass software [ERT; Brussels, Belgium]) from CT scans; and the number of patients undergoing potentially curative surgical resection. Quality of life was also assessed and will be the subject of a separate analysis.

### Statistics

Assuming a probability of a device-related serious adverse event (SAE) of 0.05, 40 participants would provide a probability of observing at least one SAE of 0.87, which is an acceptable detection rate. For the LDCR at 16 weeks, assuming a null hypothesis of 55%, an alternative hypothesis of 75%, and a significance level of 0.05 with a two-sided test, the sample size required to achieve a power of 80% was 45. The target sample size was therefore set at 45.

Three populations were defined: the intention-to-treat (ITT) population (all enrolled patients); the safety population (all enrolled patients that received any study treatment); and the per protocol (PP) population (all enrolled patients that received ^32^P microparticles). The primary endpoint is presented for the safety and PP populations. Efficacy data are presented for the ITT and PP populations.

Descriptive statistics were used. Continuous variables are presented as mean, standard deviation (SD), median, minimum, and maximum. Categorical variables are presented as frequency counts and percentages. Significance of LDCR at 16 weeks was assessed using the Fisher’s exact test by comparing the binomial proportion to the null hypothesis proportion of 0.55. Change from baseline in tumour volume, ^18^F-FDG-PET values, and CA 19-9 was assessed using the paired *t*-test. OS, LPFS, and PFS were estimated using Kaplan–Meier survival analysis.

## Results

**Figure 1:**
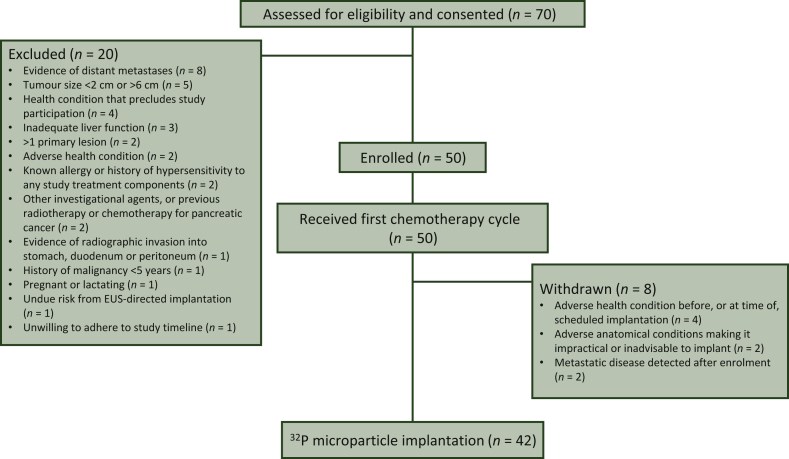
Participant flow in the PanCO study. EUS, endoscopic ultrasound.

Between March 2017 and June 2018, 50 patients were enrolled (ITT population), 50 patients received any study treatment (safety population), and 42 patients subsequently received ^32^P microparticle implantation (PP population; Figure 1) at a median (range) of 31 (21-77) days from commencing chemotherapy. Median follow-up was 31.6 months [95% confidence interval (CI): 26.5-35.1 months]. Baseline characteristics of the ITT population are summarized in Table 1. Central review of the baseline PET scans identified seven patients (5 PP) with suspected liver metastases. One patient withdrew from the study following hospitalization for TEAEs which precluded ^32^P microparticle implantation and subsequently received ^32^P microparticles off-protocol (ITT population).

**Table 1:** Baseline characteristics of the ITT population

Characteristic	*n* (%)^a^
Median (range) age, years	65 (42-84)
Sex	
Male	28 (56)
Female	22 (44)
Race	
White/Caucasian	40 (80)
Asian	7 (14)
Black/African American	3 (6)
ECOG performance status	
0	26 (52)
1	24 (48)
Pancreatic tumour location	
Head	42 (84)
Body	8 (16)
Median (range) target lesion longest diameter, cm^b^	4.5 (2.6-7.1)
Median (range) tumour volume, cc^b^	24.4 (7.9-68.7)
Median (range) CA 19-9 level, U/ml^c^	163 (1-6576)

CA, carbohydrate antigen; ECOG, Eastern Cooperative Oncology Group; ITT, intention-to-treat.

a Unless otherwise stated in left-hand column.

b By independent central reader analysis.

c *n* = 49.

### Treatment delivered

The chemotherapy received by patients in the ITT population was gemcitabine/nab-paclitaxel in 40 (80.0%) patients with a median of 4 (range, 1-25) 28-day cycles and was FOLFIRINOX in 10 (20.0%) patients with a median of 6 (range, 2-13) 14-day cycles. The median relative dose intensity was 47.0% and 41.1% in the groups receiving gemcitabine/nab-paclitaxel and FOLFIRINOX, respectively, for the first 6 or 12 cycles of chemotherapy. Chemotherapy dose reduction or delay ≥1 week occurred in 92.5% and 100.0% of participants, respectively. In the group receiving gemcitabine/nab-paclitaxel, 34 patients also received ^32^P microparticle implantation, and among these patients a pre-implantation chemotherapy dose reduction or delay of ≥1 week was observed in 26 (76.5%) patients. In the FOLFIRINOX group, eight patients also received ^32^P microparticle implantation, and among these patients a pre-implantation chemotherapy dose reduction or delay of ≥1 week was observed in seven (87.5%) patients.

Bremsstrahlung SPECT/CT imaging confirmed radiation localized to the implant site in 40 of the 42 implanted participants at 4 h post-implantation and in 36 participants at 7 days post-implantation.

### Safety and tolerability

In the ITT population, 1102 TEAEs were reported, of which 167 were grade ≥3, with 956 TEAEs (139 grade ≥3) in the PP population (Table 2). There were no treatment-related grade 5 TEAEs. The most common grade ≥3 AEs were haematological (neutropenia and anaemia) and fatigue, most of which were considered to be related to chemotherapy by the treating physician. In the PP population, 289 (30.2%) TEAEs occurred before ^32^P microparticle implantation (median follow-up: 31 days), and 667 (69.8%) occurred after ^32^P microparticle implantation (median follow-up 31 months; Supplementary Table S2, available at https://doi.org/10.1016/j.esmoop.2021.100356). A higher proportion of the PP population receiving gemcitabine/nab-paclitaxel (29/34, 85.3%) had a grade ≥3 TEAE than patients receiving FOLFIRINOX (5/8, 62.5%; Supplementary Table S3, available at https://doi.org/10.1016/j.esmoop.2021.100356).

**Table 2:** Number of patients with treatment-emergent adverse events in ≥10% of participants, by population and by attribution (possible or probable causality)

Treatment-emergent adverse event	ITT population (*n* = 50)		PP population (*n* = 42)		TEAEs attributed to ^32^P device or implantation procedure (PP population; *n* = 42)		TEAEs attributed to chemotherapy (PP population; *n* = 42)	
	All grade *n* (%)	Grade ≥3 *n* (%)	All grade *n* (%)	Grade ≥3 *n* (%)	All grade *n* (%)	Grade ≥3 *n* (%)	All grade *n* (%)	Grade ≥3 *n* (%)
Total events, *n*	1102	167	956	139	41	8	609	67
Total participants with ≥1 TEAE	50 (100.0)	41 (82.0)	42 (100.0)	34 (81.0)	16 (38.1)	3 (7.1)	42 (100.0)	28 (66.7)
Fatigue	41 (82.0)	7 (14.0)	35 (83.3)	6 (14.3)	5 (11.9)	1 (2.4)	34 (81.0)	5 (11.9)
Nausea	30 (60.0)	5 (10.0)	25 (59.5)	3 (7.1)	3 (7.1)	—	23 (54.8)	2 (4.8)
Diarrhoea	29 (58.0)	1 (2.0)	26 (61.9)	1 (2.4)	—	—	21 (50.0)	1 (2.4)
Neutropenia^a^	28 (56.0)	24 (48.0)	22 (52.4)	18 (42.9)	2 (4.8)	1 (2.4)	20 (47.6)	16 (38.1)
Abdominal pain^a^	26 (52.0)	6 (12.0)	22 (52.4)	5 (11.9)	3 (7.1)	1 (2.4)	5 (11.9)	1 (2.4)
Constipation	24 (48.0)	1 (2.0)	19 (45.2)	1 (2.4)	—	—	10 (23.8)	—
Alopecia	21 (42.0)	—	16 (38.1)	—	—	—	16 (38.1)	—
Decreased appetite	18 (36.0)	1 (2.0)	18 (42.9)	1 (2.4)	—	—	16 (38.1)	—
Vomiting	18 (36.0)	4 (8.0)	14 (33.3)	3 (7.1)	—	—	10 (23.8)	1 (2.4)
Pyrexia	17 (34.0)	3 (6.0)	16 (38.1)	3 (7.1)	—	—	11 (26.2)	2 (4.8)
Peripheral neuropathy^a^	17 (34.0)	1 (2.0)	15 (35.7)	1 (2.4)	—	—	15 (35.7)	1 (2.4)
Thrombocytopenia^a^	17 (34.0)	5 (10.0)	14 (33.3)	4 (9.5)	1 (2.4)	1 (2.4)	13 (31.0)	3 (7.1)
Anaemia^a^	15 (30.0)	7 (14.0)	14 (33.3)	7 (16.7)	1 (2.4)	—	12 (28.6)	5 (11.9)
Weight decreased	15 (30.0)	2 (4.0)	13 (31.0)	2 (4.8)	1 (2.4)	—	10 (23.8)	1 (2.4)
Rash	13 (26.0)	—	12 (28.6)	—	—	—	12 (28.6)	—
Peripheral oedema^a^	13 (26.0)	1 (2.0)	10 (23.8)	—	—	—	8 (19.0)	—
Hypokalaemia^a^	10 (20.0)	3 (6.0)	8 (19.0)	2 (4.8)	1 (2.4)	1 (2.4)	4 (9.5)	1 (2.4)
Dysgeusia	8 (16.0)	—	7 (16.7)	—	—	—	7 (16.7)	—
Hypotension	8 (16.0)	—	7 (16.7)	—	1 (2.4)	—	1 (2.4)	—
Dyspnoea	8 (16.0)	—	7 (16.7)	—	—	—	2 (4.8)	—
Pain	8 (16.0)	1 (2.0)	5 (11.9)	1 (2.4)	—	—	1 (2.4)	—
Pruritus	7 (14.0)	—	7 (16.7)	—	—	—	5 (11.9)	—
Pulmonary embolism	7 (14.0)	6 (12.0)	6 (14.3)	5 (11.9)	1 (2.4)	1 (2.4)	3 (7.1)	1 (2.4)
Mucosal inflammation	7 (14.0)	1 (2.0)	6 (14.3)	1 (2.4)	—	—	6 (14.3)	1 (2.4)
Cellulitis	6 (12.0)	1 (2.0)	6 (14.3)	1 (2.4)	—	—	4 (9.5)	—
Back pain	6 (12.0)	1 (2.0)	6 (14.3)	1 (2.4)	—	—	—	—
Paraesthesia	6 (12.0)	—	6 (14.3)	—	—	—	4 (9.5)	—
Hypomagnesemia	6 (12.0)	—	5 (11.9)	—	—	—	4 (9.5)	—
Ascites	6 (12.0)	2 (4.0)	4 (9.5)	2 (4.8)	—	—	1 (2.4)	1 (2.4)
Device occlusion (stent)	5 (10.0)	3 (6.0)	5 (11.9)	3 (7.1)	—	—	—	—
Epistaxis	5 (10.0)	1 (2.0)	5 (11.9)	1 (2.4)	—	—	4 (9.5)	—
Oral candidiasis	5 (10.0)	—	5 (11.9)	—	—	—	4 (9.5)	—
Hypoalbuminemia	5 (10.0)	4 (8.0)	4 (9.5)	3 (7.1)	1 (2.4)	1 (2.4)	3 (7.1)	2 (4.8)
Dry mouth	5 (10.0)	—	4 (9.5)	—	—	—	4 (9.5)	—
Dizziness	5 (10.0)	—	4 (9.5)	—	—	—	1 (2.4)	—

TEAEs in ≥10% of study participants at any grade (ITT or PP population). Multiple records from the same study participants are only counted once within the same category.

ITT, intention-to-treat; PP, per protocol (enrolled and implanted participants); TEAE, treatment-emergent adverse event; —, no TEAEs.

a Combined records: abdominal pain includes TEAEs reported as abdominal pain irrespective of abdominal site of pain (lower, upper, or not otherwise specified); peripheral oedema includes TEAEs reported as oedema peripheral and/or peripheral swelling; neutropenia includes TEAEs reported as neutropenia, febrile neutropenia, neutropenic sepsis, and/or neutrophil count decreased; thrombocytopenia includes TEAEs reported as thrombocytopenia and/or platelet count decreased; anaemia includes TEAEs reported as anaemia and/or haemoglobin decreased; hypokalaemia includes TEAEs reported as hypokalaemia and/or blood potassium decreased; peripheral neuropathy includes TEAEs reported as peripheral neuropathy and/or peripheral sensory neuropathy.

Of the 41 TEAEs possibly or probably related to ^32^P microparticles or implantation procedure in 16 (38.1%) patients, including 8 grade ≥3 in 3 (7.1%) patients (Table 2), 27 (5 grade ≥3) were also attributed to chemotherapy. In the PP population, 609 TEAEs were considered possibly or probably related to chemotherapy in 42 (100%) patients, including 67 grade ≥3 in 28 (66.7%) patients. Most TEAEs possibly or probably related to ^32^P microparticles occurred within 30 days of the implantation procedure and none occurred >120 days after the implantation (Supplementary Figure S1, available at https://doi.org/10.1016/j.esmoop.2021.100356). No radiation-related treatment-emergent SAEs (TESAEs) were reported. There was no evidence that the incidence of grade ≥3 TEAEs per chemotherapy cycle changed after ^32^P microparticle implantation (Supplementary Figure S2, available at https://doi.org/10.1016/j.esmoop.2021.100356).

Three treatment-emergent severe adverse device-related events (TESADEs) were reported, of which two (abdominal pain and neutropenic sepsis) occurred in the same patient. Both these TESADEs resolved and were considered possibly related to the investigational device. The third TESADE involved intravasation of the ^32^P microparticle device resulting in shunting of the implanted activity to lung. Upon review, the independent study safety review committee advised that the patient should not have been enrolled due to the presence of intratumoural varices and the exclusion criteria were strengthened to reflect this. This patient experienced no respiratory sequelae over a follow-up period of >14 months.

Twenty-six study participants had blood and urine samples taken to assess non-target ^32^P relative activity (RA). Activity was detected in the blood of 4 (15.4%) and urine of 22 (84.6%) patients. The absolute activity was below the level of quantification ≤4 h post-implantation of the ^32^P microparticle device in both blood and urine. In urine, mean activity peaked at days 7-14 (RA, 0.12%), with decreased activity (RA, 0.07%) by day 21. Mean activity in blood peaked at day 21 (RA, 0.078%) and all sampled patients had activity below the level of quantification by day 56.

### Efficacy

LDCR at 16 weeks was 82.0% (95% CI: 68.6% to 91.4%) in the ITT population and 90.5% (95% CI: 77.4% to 97.3%) in the PP population (Table 3), which met the conditions for *a priori* performance criteria.

**Table 3:** Efficacy analyses

Efficacy measure	ITT population (*N* = 50)	PP population (*N* = 42)
Local disease control^a^^,^^b^		
Patients with local disease control at 16 weeks, *n* (%)	41 (82.0)	38 (90.5)
Local disease control rate at 16 weeks (95% CI)	82.0 (68.6-91.4)	90.5 (77.4-97.3)
*P* value^c^	0.0001	<0.0001
Best tumour response by RECIST v1.1, *n* (%)^a^^,^^b^		
Complete response (CR)^d^	0	0
Partial response (PR)^d^	14 (29.8)	13 (31.0)
Stable disease^d^	31 (66.0)	29 (69.0)
Progressive disease (PD)^d^	2 (4.3)	0
Not evaluated	3	0
Objective response rate (CR + PR)^e^	14 (28.0)	13 (31.0)
Disease control rate (CR + PR + stable disease)^e^	45 (90.0)	42 (100.0)
Tumour volume response (CT scan)^a^^,^^b^		
Median (range) maximal decrease from baseline, %	−51.6 (+72.2 to −89.9)^g^	−51.9 (+11.1 to −89.9)
Mean (SD) maximal decrease from baseline, %	−44.0 (34.8)^g^	−49.1 (26.4)
*P* value^f^	<0.0001	<0.0001
Tumour response by ^18^F-FDG-PET at 12 weeks^a^^,^^b^		
Patients with evaluable images, *n*	42	39
TLG: median (range) change from baseline, %	−60.5 (+319.2 to −100.0)	−60.5 (+319.2 to −100.0)
mean (SD) change from baseline, %	−37.1 (89.9)	−34.8 (92.8)
*P* value^f^	0.0001	0.0003
SUV_Max_: median (range) change from baseline, %	−40.3 (+76.4 to −100.0)	−40.4 (+76.4 to −100.0)
mean (SD) change from baseline, %	−36.3 (43.1)	−35.8 (42.9)
*P* value^f^	<0.0001	<0.0001
SUL_Max_: median (range) change from baseline, %	−43.1 (+75.3 to −100.0)	−43.7 (+75.3 to −100.0)
mean (SD) change from baseline, %	−36.2 (46.3)	−35.9 (46.3)
*P* value^f^	0.0188	0.0232
Surgical resection with curative intent, *n* (%)	10 (20.0)	10 (23.8)
R0 margin status, *n* (% of resections)	8 (80.0)	8 (80.0)
R1 margin status, *n* (% of resections)	2 (20.0)	2 (20.0)
CA 19-9 response^b^		
Assessable patients with baseline CA 19-9 ≥35 U/ml, *n*	38	33
Median (range) maximal decrease from baseline, %	−80.7 (+50.0 to −99.9)	−82.3 (+50.0 to −99.9)
Mean (SD) maximal decrease from baseline, %	−68.1 (35.4)	−70.9 (34.0)
*P* value^f^	0.0006	0.0024
Local progression-free survival, months^a^		
Median (95% CI), uncensored for resection	9.9 (7.3-12.6)	9.8 (7.3-12.6)
Median (95% CI), patients censored before resection	9.5 (7.2-11.3)	9.3 (7.2-11.3)
Progression-free survival, months^a^		
Median (95% CI), uncensored for resection	9.3 (5.7-11.3)	9.3 (5.8-11.3)
Median (95% CI), patients censored before resection	7.7 (5.7-9.9)	7.7 (5.8-9.9)
Overall survival, months		
Median (95% CI)	15.2 (11.3-18.8)	15.5 (11.4-20.1)

CI, confidence interval; CT, computed tomography; ^18^F-FDG-PET, [^18^F]2-fluoro-2-deoxy-d-glucose positron emission tomography; ITT, intention-to-treat population; PP, per protocol population; SD, standard deviation; SUL_Max_, maximum standardized uptake value corrected for lean body mass; SUV_Max_, maximum standardized uptake value; TLG, total lesion glycolysis.

a By central image reader analysis.

b Response before surgical resection.

c *P* values for Fisher’s Exact test, comparing the binomial proportion with the null hypothesis proportion of 0.55.

d Percentages based on the number of assessable study participants.

e Percentages based on the number of all study participants.

f *P* value for paired *t*-test, percent change from baseline.

g *n* = 47 Patients with evaluable post-baseline scans.

Other measures of target tumour response showed improvement (Table 3; Supplementary Figures S3-S5, available at https://doi.org/10.1016/j.esmoop.2021.100356). Total objective response rate was 28.0% and 31.0% in the ITT and PP populations, respectively. Target tumour volume was significantly reduced compared with baseline (Table 3; Supplementary Figure S3, available at https://doi.org/10.1016/j.esmoop.2021.100356). ^18^F-FDG-PET imaging revealed significant improvements in TLG, SUL_Max_, and SUV_Max_ at 12 weeks, with a complete metabolic response in six patients (five in the PP population; Table 3; Supplementary Figure S4, available at https://doi.org/10.1016/j.esmoop.2021.100356). CA 19-9 levels were also significantly reduced (Table 3; Supplementary Figure S5, available at https://doi.org/10.1016/j.esmoop.2021.100356). In the PP population, 26 (78.8%) patients had ≥50% maximal reduction in CA 19-9 and 15 (45.5%) had a normal nadir CA 19-9.

The mean pain score at study commencement was 2.0, suggesting relatively low and/or reasonably well-controlled pain at baseline (Supplementary Figure S6, available at https://doi.org/10.1016/j.esmoop.2021.100356). Seventeen patients reported moderate-to-severe pain at baseline, defined as NRS ≥5. In this cohort, the mean change of NRS score from baseline was −3.3 before ^32^P microparticle implantation, compared with −4.7 following implantation through to end-of-study, resection, or local disease progression (median week 24).

Ten patients (all recipients of ^32^P microparticles; nine received gemcitabine/nab-paclitaxel and one received FOLFIRINOX) underwent surgical resection by Whipple procedure between 70 and 267 days after the ^32^P microparticle implantation (Table 3). Four additional patients treated with ^32^P microparticles were downstaged and technically considered for surgical resection, but could not undergo surgery due to distant metastases, comorbidities, and/or other considerations such as advanced age or patient choice. In the first 12 months following ^32^P microparticle implantation, 18 patients (36.0%) received second-line chemotherapy, 11 (22.0%) underwent surgery, and 13 (26.0%) had other treatments or procedures (Supplementary Table S4, available at https://doi.org/10.1016/j.esmoop.2021.100356).

**Figure 2:**
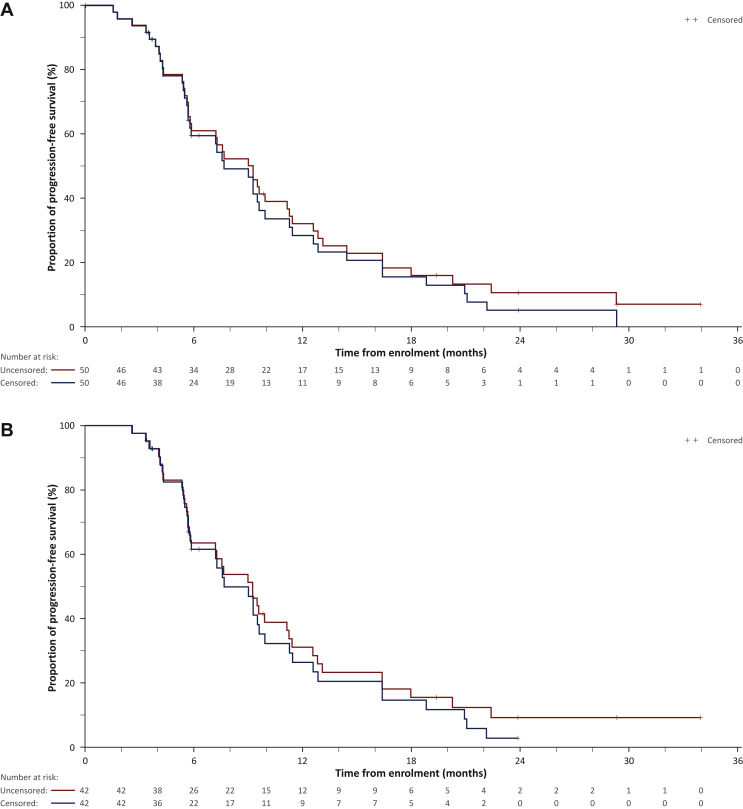

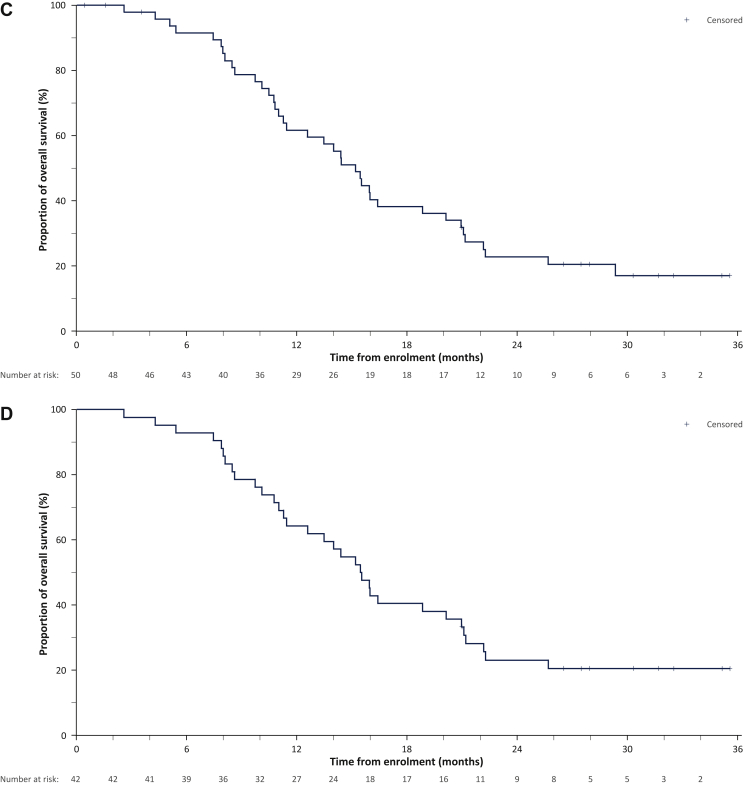
Kaplan–Meier analysis of progression-free survival at any site and overall survival. (A) Progression-free survival in the ITT population, censored or uncensored before resection. (B) Progression-free survival in the PP population, censored or uncensored before resection. (C) Overall survival in the ITT population. (D) Overall survival in the PP population. ITT, intention-to-treat; PP, per protocol (enrolled and implanted participants).

Median LPFS, PFS, and OS are summarized in Table 3 and Figure 2. Median OS was 15.2 months and 15.5 months in the ITT and PP populations, respectively.

## Discussion

The PanCO study shows that EUS-guided ^32^P microparticle implantation is feasible, with an acceptable safety profile, combined with first-line chemotherapy for LAPC over a prolonged study timeframe. This is the first published report of a prospective clinical study investigating the use of targeted intratumoural ^32^P microparticles to treat LAPC.

The overall safety profile was largely consistent with that expected in a population receiving standard-of-care chemotherapy, with no evidence suggesting significant additional risk (including the risk of radiation-related toxicity) when ^32^P microparticles were combined with contemporary systemic chemotherapy regimens.

This study also provides encouraging outcomes to suggest clinically relevant benefits for patients with unresectable LAPC treated with ^32^P microparticles combined with systemic chemotherapy. The lead secondary endpoint (LDCR at 16 weeks) was met in both the ITT and PP populations. This surrogate endpoint has been recommended in a recent consensus paper for trials of novel drug-radiotherapy combinations and may correlate with disease-free survival and OS.^23^ Even in the presence of micrometastatic disease, locoregional tumour control may translate into improvements in OS, symptom-free survival, and quality of life.^24^ Indeed, the median OS in the PP population of the PanCO study (15.5 months) compares favourably with a recent systematic literature review and meta-analysis of chemotherapy and chemoradiotherapy in LAPC (13.3 months).^4^ Individual studies (some included in the systematic review) have reported median OS in LAPC in the range of 15-19 months with induction chemotherapy using two or three agents followed by chemoradiation^25^^,^^26^ and with combination chemotherapy alone.^8^^,^^27^, ^28^, ^29^ Differences in selection criteria across studies, however, make cross-study comparisons unreliable. For example, in the PanCO study the chemotherapy regimen selection and dose reduction was at the investigators’ discretion, whereas in some other studies the delivery of the chemotherapy would be more strictly controlled and therefore the intensity of the chemotherapy regimen may vary between studies.

Beyond the local disease control, reduction in symptoms (i.e. pain) associated with tumour progression, and significant improvements from baseline in tumour volume and metabolic tumour response according to ^18^F-FDG-PET and CA 19-9, indicate a consistently encouraging response. There is no consensus on the optimum cut-off for clinically meaningful reductions in CA 19-9 in LAPC, with reductions of CA 19-9 from baseline ranging from >15% to >90% following treatment being proposed as predictors of prolonged survival in various studies.^21^^,^^30^, ^31^, ^32^, ^33^, ^34^, ^35^, ^36^ Even using the strictest of these cut-offs, an important proportion of those in the PP population of the PanCO study had significant reduction in CA 19-9 levels, and almost 50% of assessable patients had nadir CA 19-9 within the normal range.

Ten study participants had a surgical resection with curative intent following ^32^P microparticle implantation, with eight achieving an R0 resection. This represents a resection rate of 23.8% in the PP population of patients not considered surgical candidates at study enrolment. The aim of combining ^32^P-based brachytherapy with radiosensitising chemotherapy agents is to maximize the antitumoural effects in shrinking the pancreatic tumours, overall and away from the involved vessels, as well as sterilizing the surgical margins in order to increase the rate of surgical resection and the proportion with R0 margins, and minimize the risk of local recurrence. There is evidence in pancreatic cancer surgery that clear/R0 margins are associated with improved survival compared with R1 margins,^37^ and patients undergoing surgical resection had significantly better survival than patients who did not (median OS 35.3 months versus 16.3 months, respectively).^38^

Limitations include the small sample size of this pilot study and the absence of a comparator arm. Because the PanCO study was not designed to assess resection rate, the classification of patients as unresectable was left to the local multidisciplinary teams, which potentially could have led to inconsistencies among the recruited population in terms of their resectability status. The high resection rates may therefore be viewed with caution. Nevertheless, the centres involved in the study were highly experienced in managing patients with unresectable LAPC and the conversion of 23.8% of the PP population to resection does appear interesting.

The encouraging results of the PanCO study provide evidence that ^32^P microparticles can address a significant unmet clinical need in the unresectable LAPC population. Based on these results, further clinical studies are planned to assess the safety and efficacy of ^32^P microparticles in combination with standard-of-care chemotherapy regimens for LAPC, and the potential for converting unresectable patients to curative resection.
