# Supplementary material for: Results of a single-arm pilot study of 32P microparticles in unresectable locally advanced pancreatic adenocarcinoma with gemcitabine/nab-paclitaxel or FOLFIRINOX chemotherapy
Source: ESMO Open. 2021 Dec 23;7(1):100356. doi: 10.1016/j.esmoop.2021.100356 (PMC8717429; doi:10.1016/j.esmoop.2021.100356)
Supplement: Supplementary Tables and Figures [file mmc1.docx]

**Results of a Single-Arm Pilot Study of ^32^P Microparticles in Unresectable Locally Advanced Pancreatic Adenocarcinoma with Gemcitabine/Nab-Paclitaxel or FOLFIRINOX Chemotherapy**

*P. J. Ross, H. S. Wasan, D. Croagh, M. Nikfarjam, N. Nguyen, M. Aghmesheh, A. M. Nagrial, D. Bartholomeusz, A. Hendlisz, T. Ajithkumar, C. Iwuji, N. E. Wilson, D. M. Turner, D.* *C. James, E. Young, M. T. Harris.*

https://doi.org/10.1016/j.esmoop.2021.100356

**Supplementary information**

**Figure S1.** Timing of treatment-emergent adverse events (TEAEs) attributed to ^32^P microparticles or implantation procedure, or chemotherapy (PP population), adjusted to the day of ^32^P microparticle implantation. PP, per protocol (enrolled and implanted participants); TEAE, treatment-emergent adverse event.

**Figure S2.** Incidence of grade ≥3 treatment-emergent adverse event (TEAE) categories by chemotherapy cycle (PP population), adjusted to the day of ^32^P microparticle implantation. Percentages are the proportion of the total population per chemotherapy cycle. Multiple records from the same study participant are only counted once within the same category. Neutropenia includes TEAEs reported as neutropenia, febrile neutropenia, neutropenic colitis, neutropenic sepsis and/or neutrophil count decreased. Eight of the 42 study participants in the PP cohort received 2 cycles of chemotherapy prior to ^32^P microparticle implantation. PP, per protocol (enrolled and implanted participants); TEAE, treatment-emergent adverse event.

**Figure S3.** Target tumour volume response per patient: maximal percentage decrease from baseline. All measurements prior to surgical resection. Outcomes colour coded by cohort (no ^32^P implant versus per protocol population [PP]), RECIST response and subsequent surgical resection. PR, partial response; SD, stable disease.

**Figure S4.** FDG-PET target tumour response per patient: Percentage change at 12 weeks from baseline of (A) Total Lesion Glycolysis (TLG); (B) Maximum Standard Uptake Value (SUV_Max_); and (C) Maximum Standard Uptake Value normalized to lean body mass (SUL_Max_). All measurements prior to surgical resection. Bars capped at 100% increase (values included). Outcomes colour coded by cohort (no ^32^P implant versus per protocol), RECIST response and subsequent surgical resection. FDG-PET, fluorodeoxyglucose positron emission tomography; PP, per protocol population; PR, partial response; SD, stable disease.

**Figure S5.** CA 19-9 response per patient (with baseline >35 U/ml): maximal percentage decrease from baseline. All measurements prior to surgical resection. Outcomes colour coded by cohort (no ^32^P implant versus per protocol population [PP]), RECIST response and subsequent surgical resection. CA, carbohydrate antigen; PR, partial response; SD, stable disease.

**Figure S6.** Mean pain scores over time, measured by Numerical Rating Score (NRS), overall and for the subgroups of patients presenting with either mild (NRS <5; range 0 to 3) or moderate-to-severe pain (NRS ≥5; range 5 to 10) at baseline. All measurements prior to surgical resection or local disease progression. ITT, intention-to-treat.

**Table S1**. Inclusion and exclusion criteria for the PanCO study

| **Inclusion criteria** | **Exclusion criteria** |
| --- | --- |
| - ≥18 years old - Histologically or cytologically proven, unresectable locally advanced pancreatic carcinoma - Target tumour diameter 2–6 cm - ECOG performance status 0 or 1 - Adequate renal function (serum creatinine <1.5 times the ULN) - Adequate liver function (serum liver transaminases ≤3 times ULN and serum bilirubin ≤1.5 times ULN) - Adequate bone marrow function (white blood cell count ≥3000/mm^3^, absolute neutrophil count ≥1500/mm^3^, haemoglobin ≥9 g/dl, and platelets ≥100,000/mm^3^) - Life expectancy of ≥3 months at screening - Not pregnant and using adequate birth control if of child-bearing potential | - Evidence of distant metastases based on CT scan - Evidence of radiographic invasion into the stomach, duodenum, or peritoneum - More than one primary lesion - Any previous radiotherapy or chemotherapy for pancreatic cancer - Use of any investigational agent within the last 30 days - Unacceptable risks for EUS-directed implantation according to the investigator - History of malignancy in the last 5 years - A known allergy to any of the components of the test device |

CT, computed tomography; ECOG, Eastern Cooperative Oncology Group; EUS, endoscopic ultrasound; ULN, upper limit of normal.

**Table S2**. Incidence of treatment-emergent adverse events before and after ^32^P microparticle implantation (PP population)

| Treatment-emergent  adverse event ^a^ | Pre-implantation (n=42) | | Post-implantation (n=42) | |
| --- | --- | --- | --- | --- |
|  | (median follow-up, 1 month) | | (median follow-up, 31 months) | |
|  | All grade  n (%) | Grade ≥3  n (%) | All grade  n (%) | Grade ≥3  n (%) |
| Total events, n | 289 | 25 | 667 | 114 |
| Total participants with ≥1 TEAE | 41 (97.6) | 18 (42.9) | 42 (100.0) | 29 (69.0) |
| Fatigue | 24 (57.1) | 2 (4.8) | 21 (50.0) | 4 (9.5) |
| Nausea | 16 (38.1) | - | 18 (42.9) | 3 (7.1) |
| Diarrhoea | 14 (33.3) | - | 21 (50.0) | 1 (2.4) |
| Neutropenia ^b^ | 12 (28.6) | 8 (19.0) | 14 (33.3) | 11 (26.2) |
| Abdominal pain ^b^ | 6 (14.3) | 1 (2.4) | 19 (45.2) | 4 (9.5) |
| Constipation | 9 (21.4) | - | 13 (31.0) | 1 (2.4) |
| Alopecia | 13 (31.0) | - | 4 (9.5) | - |
| Decreased appetite | 6 (14.3) | - | 16 (38.1) | 1 (2.4) |
| Vomiting | 7 (16.7) | 1 (2.4) | 8 (19.0) | 3 (7.1) |
| Pyrexia | 4 (9.5) | 1 (2.4) | 13 (31.0) | 2 (4.8) |
| Peripheral neuropathy ^b^ | 4 (9.5) | - | 12 (28.6) | 1 (2.4) |
| Thrombocytopenia ^b^ | 5 (11.9) | - | 13 (31.0) | 4 (9.5) |
| Anaemia ^b^ | 2 (4.8) | 1 (2.4) | 14 (33.3) | 7 (16.7) |
| Weight decreased | 6 (14.3) | - | 13 (31.0) | 2 (4.8) |
| Rash | 11 (26.2) | - | 4 (9.5) | - |
| Peripheral oedema ^b^ | 1 (2.4) | - | 8 (19.0) | - |
| Hypokalaemia ^b^ | 1 (2.4) | - | 8 (19.0) | 2 (4.8) |
| Dysgeusia | 4 (9.5) | - | 5 (11.9) | - |
| Hypotension | - | - | 7 (16.7) | - |
| Dyspnoea | 2 (4.8) | - | 6 (14.3) | - |
| Pain | 4 (9.5) | - | 1 (2.4) | 1 (2.4) |
| Pruritus | 3 (7.1) | - | 4 (9.5) | - |
| Pulmonary embolism | - | - | 6 (14.3) | 5 (11.9) |
| Mucosal inflammation | 4 (9.5) | - | 3 (7.1) | 1 (2.4) |
| Cellulitis | 5 (11.9) | 1 (2.4) | 3 (7.1) | - |
| Back pain | 4 (9.5) | 1 (2.4) | 3 (7.1) | - |
| Paraesthesia | 3 (7.1) | - | 4 (9.5) | - |
| Hypomagnesemia | 2 (4.8) | - | 4 (9.5) | - |
| Ascites | 1 (2.4) | - | 3 (7.1) | 2 (4.8) |
| Device occlusion (stent) | 1 (2.4) | 1 (2.4) | 4 (9.5) | 2 (4.8) |
| Epistaxis | 3 (7.1) | - | 2 (4.8) | 1 (2.4) |
| Oral candidiasis | - | - | 5 (11.9) | - |
| Hypoalbuminemia | - | - | 4 (9.5) | 3 (7.1) |
| Dry mouth | 1 (2.4) | - | 3 (7.1) | - |
| Dizziness | 2 (4.8) | - | 2 (4.8) | - |

Notes: TEAEs in ≥10% of study participants at any grade (PP population). Multiple records from the same study participant are only counted once within the same category.

PP, per protocol (enrolled and implanted participants); TEAE, treatment-emergent adverse event; - no TEAEs.

^a^ Presented in the same order as Table 2 from main manuscript.

^b^ Combined records: Abdominal pain includes TEAEs reported as abdominal pain irrespective of abdominal site of pain (lower, upper or not otherwise specified); Peripheral oedema includes TEAEs reported as oedema peripheral and/or peripheral swelling; Neutropenia includes TEAEs reported as neutropenia, febrile neutropenia, neutropenic colitis, neutropenic sepsis and/or neutrophil count decreased; Thrombocytopenia includes TEAEs reported as thrombocytopenia and/or platelet count decreased; Anaemia includes TEAEs reported as anaemia and/or haemoglobin decreased; Hypokalaemia includes TEAEs reported as hypokalaemia and/or blood potassium decreased; Peripheral neuropathy includes TEAEs reported as peripheral neuropathy and/or peripheral sensory neuropathy.

**Table S3.** Incidence of treatment-emergent adverse events by chemotherapy regimen group (PP population)

| Treatment-emergent  adverse event ^a^ | Gemcitabine/nab-paclitaxel (n=34) | | FOLFIRINOX (n=8) | |
| --- | --- | --- | --- | --- |
|  | All grade  n (%) | Grade ≥3  n (%) | All grade  n (%) | Grade ≥3  n (%) |
| Total events, n | 779 | 123 | 177 | 16 |
| Total participants with ≥1 TEAE | 34 (100.0) | 29 (85.3) | 8 (100.0) | 5 (62.5) |
| Fatigue | 29 (85.3) | 4 (11.8) | 6 (75.0) | 2 (25.0) |
| Nausea | 20 (58.8) | 3 (8.8) | 5 (62.5) | - |
| Diarrhoea | 19 (55.9) | - | 7 (87.5) | 1 (12.5) |
| Neutropenia ^b^ | 18 (52.9) | 17 (50.0) | 4 (50.0) | 1 (12.5) |
| Abdominal pain ^b^ | 19 (55.9) | 3 (8.8) | 3 (37.5) | 2 (25.0) |
| Constipation | 17 (50.0) | 1 (2.9) | 2 (25.0) | - |
| Alopecia | 16 (47.1) | - | - | - |
| Decreased appetite | 15 (44.1) | 1 (2.9) | 3 (37.5) | - |
| Vomiting | 11 (32.4) | 3 (8.8) | 3 (37.5) | - |
| Pyrexia | 13 (38.2) | 1 (2.9) | 3 (37.5) | 2 (25.0) |
| Peripheral neuropathy ^b^ | 11 (32.4) | 1 (2.9) | 4 (50.0) | - |
| Thrombocytopenia ^b^ | 12 (35.3) | 4 (11.8) | 2 (25.0) | - |
| Anaemia ^b^ | 12 (35.3) | 6 (17.6) | 2 (25.0) | 1 (12.5) |
| Weight decreased | 11 (32.4) | 2 (5.9) | 2 (25.0) | - |
| Rash | 10 (29.4) | - | 2 (25.0) | - |
| Peripheral oedema ^b^ | 9 (26.5) | - | 1 (12.5) | - |
| Hypokalaemia ^b^ | 6 (17.6) | 2 (5.9) | 2 (25.0) | - |
| Dysgeusia | 7 (20.6) | - | - | - |
| Hypotension | 5 (14.7) | - | 2 (25.0) | - |
| Dyspnoea | 6 (17.6) | - | 1 (12.5) | - |
| Pain | 5 (14.7) | 1 (2.9) | - | - |
| Pruritus | 5 (14.7) | - | 2 (25.0) | - |
| Pulmonary embolism | 5 (14.7) | 4 (11.8) | 1 (12.5) | 1 (12.5) |
| Mucosal inflammation | 5 (14.7) | 1 (2.9) | 1 (12.5) | - |
| Cellulitis | 6 (17.6) | 1 (2.9) | - | - |
| Back pain | 4 (11.8) | - | 2 (25.0) | 1 (12.5) |
| Paraesthesia | 4 (11.8) | - | 2 (25.0) | - |
| Hypomagnesemia | 4 (11.8) | - | 1 (12.5) | - |
| Ascites | 3 (8.8) | 2 (5.9) | 1 (12.5) | - |
| Device occlusion (stent) | 4 (11.8) | 3 (8.8) | 1 (12.5) | - |
| Epistaxis | 5 (14.7) | 1 (2.9) | - | - |
| Oral candidiasis | 2 (5.9) | - | 3 (37.5) | - |
| Hypoalbuminemia | 3 (8.8) | 2 (5.9) | 1 (12.5) | 1 (12.5) |
| Dry mouth | 3 (8.8) | - | 1 (12.5) | - |
| Dizziness | 3 (8.8) | - | 1 (12.5) | - |

Notes: TEAEs in ≥10% of study participants at any grade (PP Cohort). Multiple records from the same study participant are only counted once within the same category.

PP, per protocol (enrolled and implanted participants); TEAE, treatment-emergent adverse event; - no TEAEs.

^a^ Presented in the same order as Table 2 from main manuscript.

^b^ Combined records: Abdominal pain includes TEAEs reported as abdominal pain irrespective of abdominal site of pain (lower, upper or not otherwise specified); Peripheral oedema includes TEAEs reported as oedema peripheral and/or peripheral swelling; Neutropenia includes TEAEs reported as neutropenia, febrile neutropenia, neutropenic colitis, neutropenic sepsis and/or neutrophil count decreased; Thrombocytopenia includes TEAEs reported as thrombocytopenia and/or platelet count decreased; Anaemia includes TEAEs reported as anaemia and/or haemoglobin decreased; Hypokalaemia includes TEAEs reported as hypokalaemia and/or blood potassium decreased; Peripheral neuropathy includes TEAEs reported as peripheral neuropathy and/or peripheral sensory neuropathy.

**Table S4**. Subsequent treatments in the first 12 months following ^32^P microparticle implantation

| **Treatment, n (%)** | **Patients receiving**  **1^st^-line gemcitabine/nab-paclitaxel** | | **Patients receiving**  **1^st^-line FOLFIRINOX** | | **All Patients** | |
| --- | --- | --- | --- | --- | --- | --- |
|  | **ITT population** (n=40) | **PP** **population** (n=34) | **ITT** **population** (n=10) | **PP** **population** (n=8) | **ITT** **population** (n=50) | **PP** **population** (n=42) |
| **Adjuvant chemotherapy post-resection** | **6 (15.0%)** | **6 (17.6%)** | **-** | **-** | **6 (60.0%)** | **6 (14.3%)** |
| Gemcitabine/nab-paclitaxel | 5 (12.5%) | 5 (14.7%) | - | - | 5 (10.0%) | 5 (11.9%) |
| Gemcitabine + capecitabine | 1 (2.5%) | 1 (2.9%) | - | - | 1 (2.0%) | 1 (2.4%) |
|  |  |  |  |  |  |  |
| **2^nd^-line chemotherapy** | **13 (32.5%)** | **11 (32.4%)** | **5 (50.0%)** | **5 (62.5%)** | **18 (36.0%)** | **16 (38.1%)** |
| Liposomal irinotecan + 5FU/LV | 3 (7.5%) | 3 (8.8%) | - | - | 3 (6.0%) | 3 (7.1%) |
| FOLFIRI | 2 (5.0%) | 2 (5.9%) | - | - | 2 (4.0%) | 2 (4.8%) |
| FOLFOX | 2 (5.0%) | 1 (2.9%) | - | - | 2 (4.0%) | 1 (2.4%) |
| Panitumumab | 1 (2.5%) | 1 (2.9%) | - | - | 1 (2.0%) | 1 (2.4%) |
| Nivolumab + PG545 | 1 (2.5%) | 1 (2.9%) | - | - | 1 (2.0%) | 1 (2.4%) |
| Gemcitabine/nab-paclitaxel | - | - | 4 (40.0%) | 4 (50.0%) | 4 (8.0%) | 4 (9.5%) |
|  |  |  |  |  |  |  |
| **3^rd^-line chemotherapy** | **1 (2.5%)** | **1 (2.9%)** | **1 (10.0%)** | **1 (12.5%)** | **2 (4.0%)** | **2 (4.8%)** |
| FOLFIRINOX | 1 (2.5%) | 1 (2.9%) | - | - | 1 (2.0%) | 1 (2.4%) |
| Oxaliplatin + FP | - | - | 1 (10.0%) | 1 (12.5%) | 1 (2.0%) | 1 (2.4%) |
|  |  |  |  |  |  |  |
| **Surgical procedures** | **10 (25.0%)** | **10 (29.4%)** | **1 (10.0%)** | **1 (12.5%)** | **11 (22.0%)** | **11 (26.2%)** |
| Surgical resection (pancreaticoduodenectomy) | 9 (22.5%) | 9 (26.5%) | 1 (10.0%) | 1 (12.5%) | 10 (20.0%) | 10 (23.8%) |
| Exploratory laparotomy | 1 (2.5%) | 1 (2.9%) | - | - | 1 (2.0%) | 1 (2.4%) |
|  |  |  |  |  |  |  |
| **Other procedures/interventions** | **9 (22.5%)** | **7 (20.6%)** | **4 (40.0%)** | **2 (25.0%)** | **13 (26.0%)** | **9 (21.4%)** |
| External beam radiotherapy | 7 (17.5%) | 6 (17.6%) | 2 (20.0%) | 1 (12.5%) | 9 (18.0%) | 7 (16.7%) |
| OncoSil™ (off protocol) | 1 (2.5%) | 1 (2.9%) | - | - | 1 (2.0%) | 1 (2.9%) |
| Irreversible electroporation | - | - | 1 (10.0%) | - | 1 (2.0%) | - |
| Radiofrequency ablation | - | - | 1 (10.0%) | 1 (12.5%) | 1 (2.0%) | 1 (2.4%) |
|  |  |  |  |  |  |  |

FOLFIRI, irinotecan + 5FU/LV; FOLFOX, oxaliplatin + 5FU/LV ; FP, fluoropyrimidine; ITT, intention-to-treat; LV, leucovorin; PG545, pixatimod; PP, per protocol (enrolled and implanted participants); 5FU, 5-fluorouracil.
